# ﻿Description of a new species of the *Petrolisthesgalathinus* complex from the Caribbean Sea, and resurrection of *Petrolisthesoccidentalis* from the East Pacific (Crustacea, Anomura, Porcellanidae)

**DOI:** 10.3897/zookeys.1191.111570

**Published:** 2024-02-16

**Authors:** Alexandra Hiller, Bernd Werding

**Affiliations:** 1 Smithsonian Tropical Research Institute, Apartado 0843–03092, Panama, Panama Smithsonian Tropical Research Institute Panama Panama; 2 Senckenberg Research Institute and Natural History Museum, Frankfurt am Main, Germany Senckenberg Research Institute and Natural History Museum Frankfurt am Main Germany

**Keywords:** Caribbean, colour morphs, ecological range, geographical range, *Petrolisthescoeruleus* sp. nov., *Petrolisthesoccidentalis*

## Abstract

The *Petrolisthesgalathinus* complex currently consists of six American species distributed in the West Atlantic, including the amphi-American *P.galathinus*. All species in the complex are similar in their adult morphology but differ in colour, size, larval morphology, and shape of the adult sternal plate. The West Atlantic species have different geographic ranges, which overlap in the southern Caribbean. Previously published molecular data support the monophyly of the complex, and the reciprocal monophyly of each described species and further clades corresponding to different colour morphs. Here, the morph *P.caribensis* “Blue” is described as *Petrolisthescoeruleus***sp. nov.**, and *Petrolisthesoccidentalis* is formally resurrected for the Pacific individuals of *P.galathinus*. By adding these two species to the *P.galathinus* complex, this now consists of eight species. Colour illustrations of all species and colour morphs are provided and their geographic distributions and ecological ranges are discussed and updated.

## ﻿Introduction

**Figure 1.**
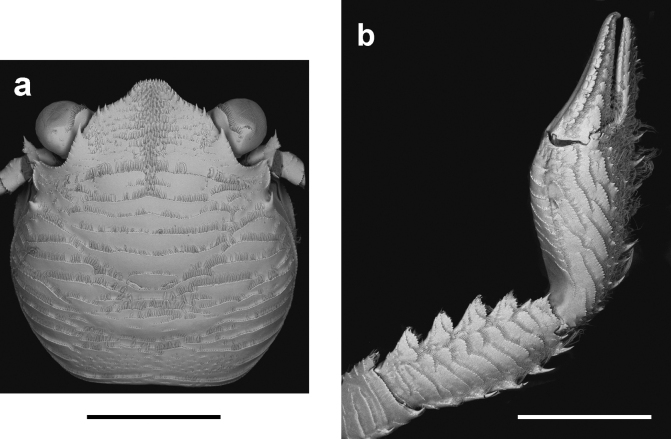
Scanning Electron Microscopy (SEM) image of **a** carapace and **b** right cheliped of *Petrolisthescaribensis* Werding, a member of the *Petrolisthesgalathinus* complex. Scale bars: 0.2 cm.

**Figure 2.**
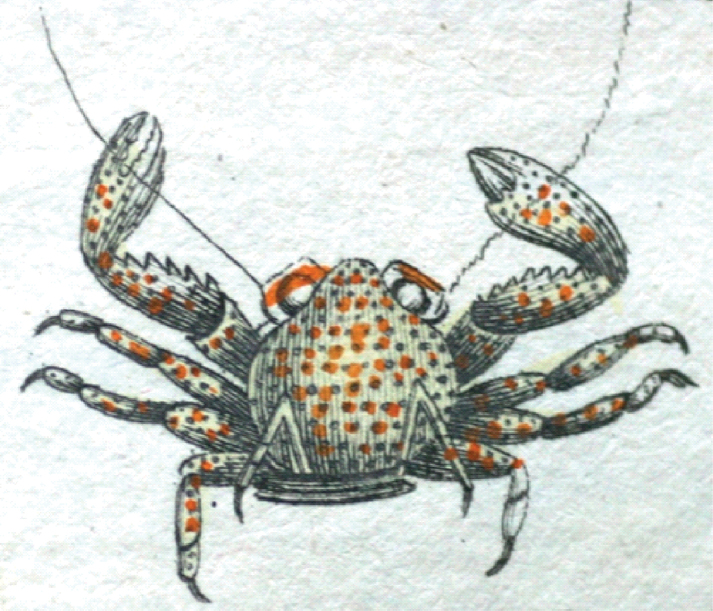
*Petrolisthesgalathinus* (Bosc), original drawing by Bosc (1802: pl. 6, fig. 2).

*Petrolisthesgalathinus* (Bosc, 1802) is an American species, described as *Porcellanagalathina* because of the transverse piliferous ridges covering carapace and extremities resembling those of the galatheid genus *Galathea* (Fig. 1). For more than two centuries the species remained subject of taxonomic conflict for three main reasons: Bosc´s ambiguous description failed to mention a type locality, which was declared as unknown (“on ignore son pays natal”; Bosc 1802: 233), the drawing depicting his holotype specimen consists of a crude sketch (see Fig. 2), and type material does not seem to be traceable. This ambiguous description prompted a labyrinthic path to recreate the morphology and origin of Bosc´s *P.galathinus*. Gibbes (1854), amongst others, criticized Bosc´s drawing because it exhibited “dots instead of stripes”, a pattern which did not correspond to observations by earlier authors (e.g., Benedict 1901). Gibbes´ remarks also alluded to different colour patterns, morphologically matching Bosc´s description. Based on specimens from Puerto Rico, Benedict (1901) described two colour varieties, one with purple stripes matching Gibbes´ (1854) specimens, and another with a double white cross on the carapace. Decades later, Rickner (1975) emphasised the considerable colour variation of specimens from the eastern coast of Mexico, and Williams (1984) reconstructed the history of different colour forms described by previous authors.

Regarding the possible type locality of *P.galathinus*, Latreille (1803: 76) declared the Antilles as the original locality of the species: “Elle se trouve aux Antilles”. Later mentions further referred to its provenance: Desmarest (1825) mentioned Georgia and Florida, and Gray (1831) reported specimens from the British Museum as coming from “North America”. Gibbes (1850) described *Porcellanasexspinosa*, and in a later study (Gibbes 1854), based on own collections from Key West, Florida, concluded that his species was synonymous with Bosc´s, and that it also occurred further north, up to South Carolina. In this study Gibbes (1854) reviewed the literature, acknowledging two western Atlantic species with the typical transverse piliferous rugae: *Porcellagalathina* Bosc from the southern Atlantic coast of the United States, and *Porcellanaboscii*? Savigny sensu Dana (1852) from Brazil. Gibbes concluded that the specimens from Brazil were different from Savigny´s *P.boscii* Audouin, 1826 from Egypt (de Savigny 1809) and described *Porcellanadanae* Gibbes, 1854. *Porcellanaboscii* was only listed by de Savigny (1809) but was later described by Audouin (1826). The species is currently accepted as *Petrolisthesboscii* (Audouin) from the Indo-West Pacific (see Werding and Hiller 2007).

In the decades following Bosc´s description, West Atlantic species of *Petrolisthes* with piliferous transverse ridges on carapace and extremities were reported as *P.galathinus* from localities throughout the Caribbean and southwards to Brazil. The eastern Pacific individuals, morphologically matching *P.galathinus*, were described as *Petrolisthesoccidentalis* Stimpson, 1859, based on specimens collected in Panama. However, Boone (1931) and Schmitt (1935) reported specimens from the East Pacific under the name *P.galathinus*.

In her study on the Porcellanidae of the western North Atlantic, Haig (1956) extensively reviewed the literature and synonymy of *Petrolisthesgalathinus*, concluding that West Atlantic and East Pacific specimens should be considered as one species. As a consequence, all subsequent authors (e.g. Chace 1956; Haig 1962, 1966; Fausto-Filho 1968; Gore 1970, 1974; Rouse 1970; Coelho and Ramos 1972; Rickner 1975; Gore and Abele 1976; Hiller et al. 2004) treated *P.galathinus* as a single widespread species distributed throughout the western Atlantic from North Carolina (U.S.A.) to Santa Catarina (Brazil), and in the eastern Pacific from Jalisco (Mexico) to Ecuador (Hiller et al. 2004).

**Figure 3.**
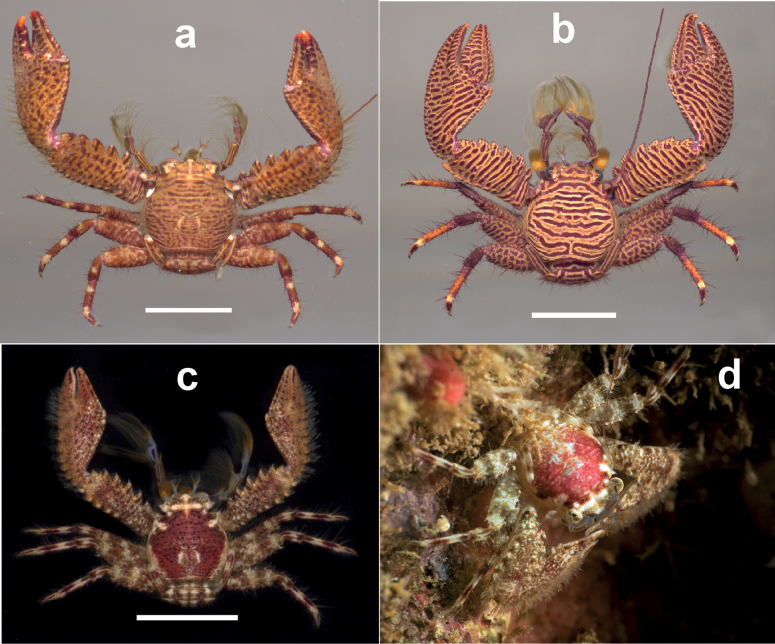
Dorsal view of **a***Petrolisthesrosariensis*, male, Islas del Rosario, Colombian Caribbean **b***P.columbiensis*, female, Islas del Rosario, Colombian Caribbean **c***P.caribensis*, male, Islas del Rosario, Colombian Caribbean **d***P.caribensis*, Roatán, Honduras, photo courtesy of M. Charteris. Scale bars: 0.6 cm (**a**); 0.8 cm (**b**); 0.3 cm (**c**).

**Figure 4.**
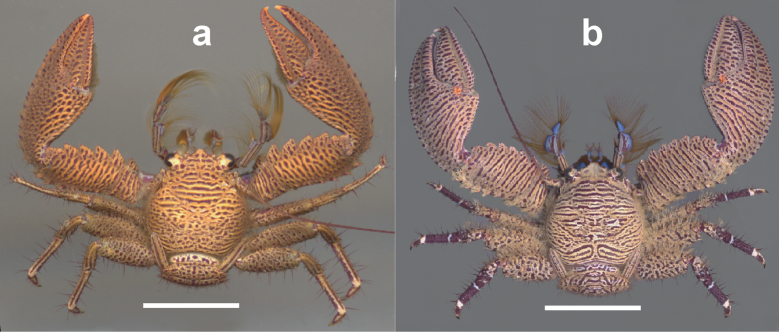
Dorsal view of **a***Petrolisthessanmartini*, male, Islas del Rosario, Colombian Caribbean **b***P.bolivarensis*, male, Islas del Rosario, Colombian Caribbean. Scale bars: 0.6 cm (**a**); 1.7 cm(**b**).

**Figure 5.**
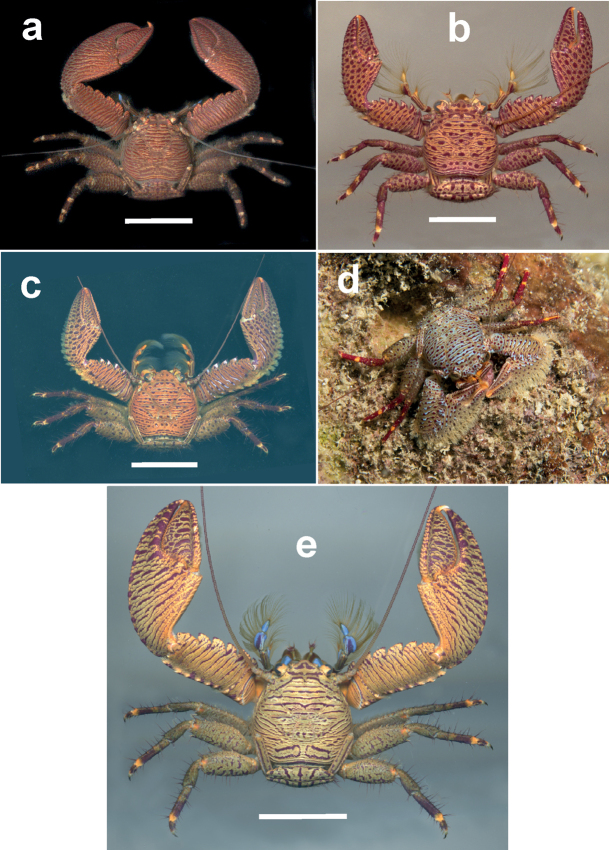
Dorsal view of colour morphs within *Petrolisthesgalathinus*, as designated by Hiller et al. (2006)**a** East Pacific morph, here resurrected as *P.occidentalis* Stimpson, male, Naos Island, Panamanian Pacific **b***P.galathinus* “Spots”, male, Islas del Rosario, Colombian Caribbean **c***P.galathinus* “White Teeth”, female (ov), Isla Cubagua, Venezuela **d** “White Teeth”, Roatán, Honduras, photo courtesy of M. Charteris **e***P.galathinus* “Stripes”, male, Gulf of Morrosquillo, Colombian Caribbean. Scale bars: 1.65 cm (**a**); 0.71 cm (**b**); 1.36 cm(**c**); 1.35 cm(**e**).

Werding (1977) recognised different species in the Colombian Caribbean, later describing *P.rosariensis* Werding, 1982 (Fig. 3a). Werding (1983) concluded that *P.galathinus* comprised a complex of several species and described *P.columbiensis*Werding (1983) (Fig. 3b) and *P.caribensis*Werding (1983) (Fig. 3c, d), only distinguishable from *P.galathinus* by their different colouration and two discrete morphological characters (Table 1). Later collections in the Colombian Caribbean revealed the presence of other colour types, described as *P.sanmartini* Werding & Hiller (2002) (Fig. 4a) and *P.bolivarensis*, Werding & Kraus (2003) (Fig. 4b), reaching a total of six species, including *P.galathinus*. Hiller et al. (2006) reconstructed the phylogenetic history of the complex based on mitochondrial DNA sequences and concluded that all species were reciprocally monophyletic, and that further clades, taxonomically matching *P.galathinus* (Fig. 5a–e), supported the presence of new species.

**Table 1.** Morphotypes and distinguishing characters of the *Petrolisthesgalathinus* complex.

**Species**	**Number of epibranchial spines**	**Number of spines on inner border of dactylus of all walking legs**	**Maximum size of male adults (carapace width in mm)**
*P.bolivarensis* Werding & Kraus	1	3	> 15.0
*P.caribensis* Werding	1	4	9.4
*P.coeruleus* sp. nov.	1	4	12.4
*P.columbiensis* Werding	2	4	7.2
*P.galathinus* (Bosc) “Stripes–Spots”	1	3	> 14.0
*P.galathinus* (Bosc) “White Teeth”	1	3	> 15.0
*P.occidentalis* Stimpson	1	3	> 17.0
*P.rosariensis* Werding	2	4	6.0
*P.sanmartini* Werding & Hiller	1	5	6.5

**Figure 6.**
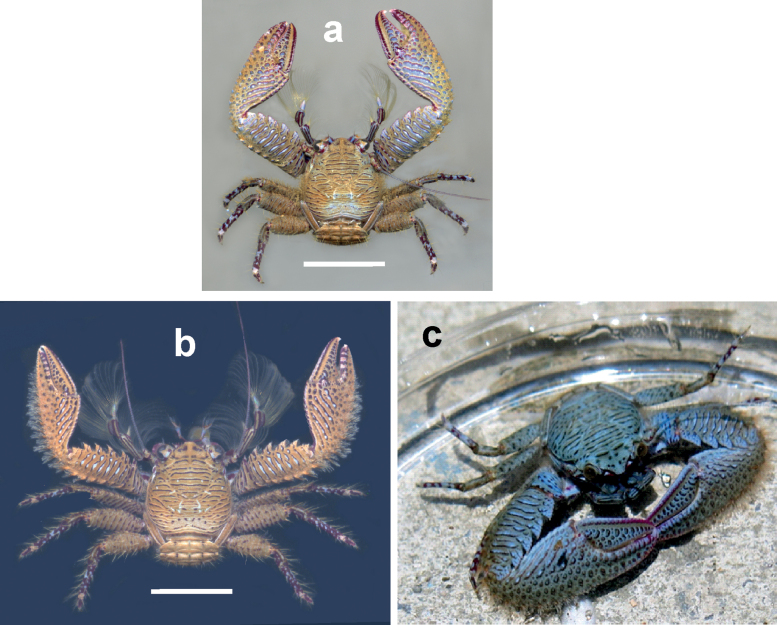
Dorsal view of *Petrolisthescoeruleus* sp. nov. **a** male, Islas del Rosario, Colombian Caribbean **b** male, Islas del Rosario, Colombian Caribbean **c** Bocas del Toro, Panamanian Caribbean, photograph courtesy of T. Deuss. Scale bars: 0.5 cm (**a**); 0.65 cm (**b**).

**Figure 7.**
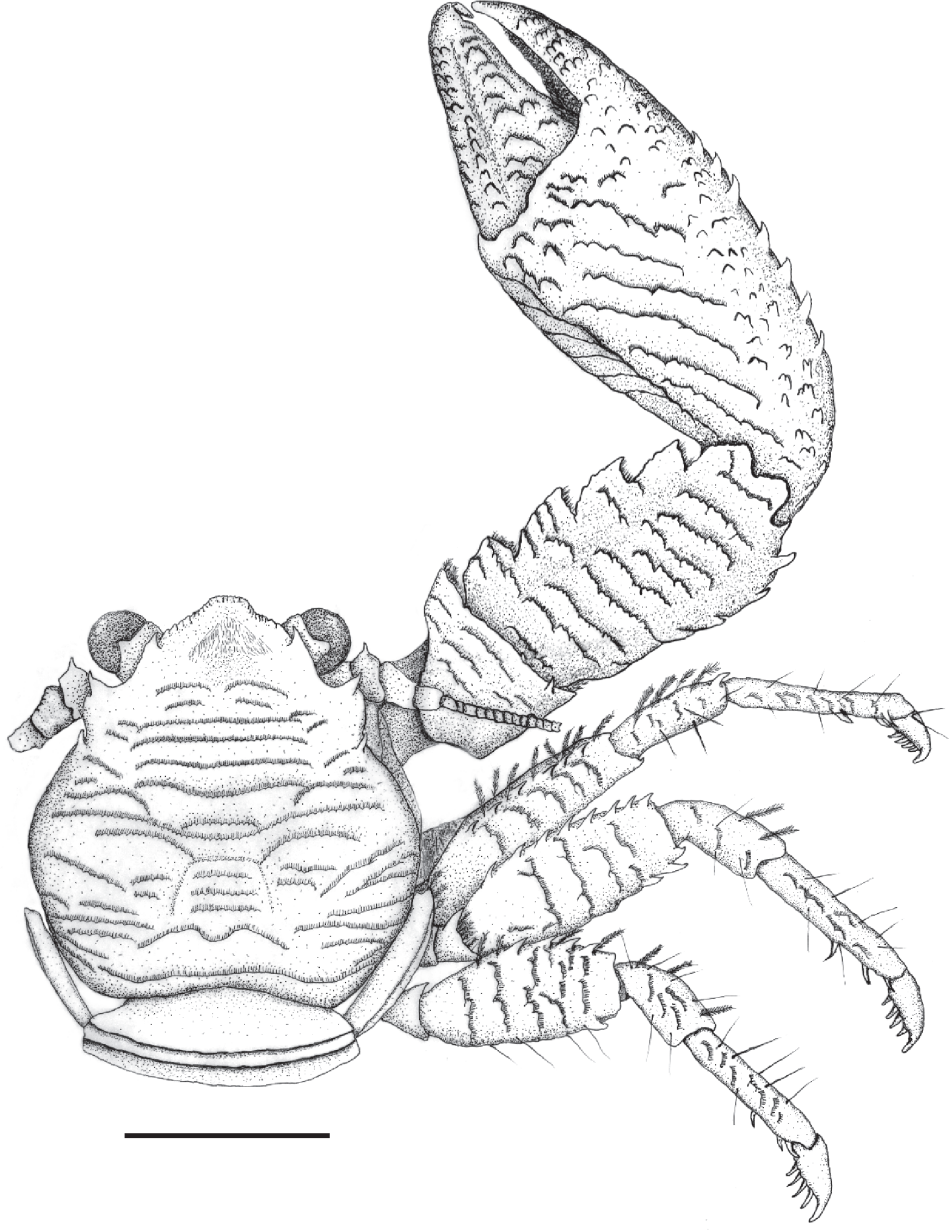
Dorsal view of *Petrolisthescoeruleus* sp. nov., male, Punta Galeta, Colón, Panamanian Caribbean. Setae on outer margin of cheliped manus omitted to depict spines. Scale bar: 0.4 cm.

**Figure 8.**
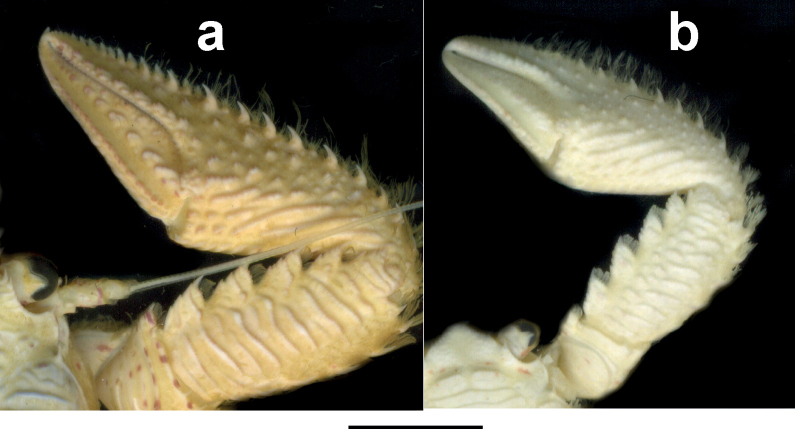
Dorsal view of right cheliped of **a***Petrolisthescoeruleus* sp. nov. and **b***P.caribensis*. Scale bars: 5 mm (**a, b**). Specimens preserved in ethanol.

Here, we describe *P.coeruleus* sp. nov. (Figs 6a–c, 7, 8a), which corresponds to the “Blue” morph revealed in the phylogeny by Hiller et al. (2006), and resurrect *P.occidentalis* Stimpson, 1859 (Fig. 5a) for the Eastern Pacific individuals of *P.galathinus*. We update the ecological and geographic information of all members of the complex and address the most plausible identity of Bosc´s *P.galathinus*.

## ﻿Material and methods

Material of *Petrolisthescoeruleus* sp. nov. collected in Belize and the Panamanian and Colombian Caribbean, and of *P.occidentalis* collected in the Panamanian East Pacific, was used for morphological examination. Type material of the new species was deposited in the Senckenberg Naturmuseum Frankfurt (**SMF**), Germany, and the Museo de Peces de Agua Dulce e Invertebrados (**MUPADI**) de la Universidad Autónoma de Chiriquí, Panamá (**UNACHI**). Type specimens of *P.occidentalis* are deposited in the Museum of Comparative Zoology (**MCZ**), Harvard University, USA. Additional material of the new species and of *P.occidentalis* was deposited in the collections of the MUPADI in Panama. We updated the geographic and ecological ranges of all species of the *P.galathinus* complex based on new records of material collected by the authors and material found in the Florida Museum of Natural History (**FMNH**). Measurements of specimens are given in mm and correspond to carapace length, followed by carapace width.

## ﻿Results

### ﻿Systematic account

**Family Porcellanidae Haworth, 1825: 184.**

**Genus *Petrolisthes* Stimpson, 1858: 227.**

#### ﻿ Petrolisthes coeruleus sp. nov.

Taxon classificationAnimaliaDecapodaPorcellanidae

8089FF36-36B4-5EAD-90BA-4700502F542D

https://zoobank.org/1E5A0FF9-CD71-425D-87BB-439771E73B59

Figs 6a–c , 7 , 8a

Petrolisthes galathinus Werding, 1982: 443 (part, Islas del Rosario).
Petrolisthes caribensis “Blue”: Hiller et al. 2006: 552.

##### Type material examined.

***Holotype***: Male, MUPADI–Crus–14, West Atlantic, Panamá, Colón, Punta Galeta, 9°24.161'N, 79°51.634'W; in *Poritesporites* (Pallas, 1766), 0.5 m, leg. A Hiller, 12 Apr. 2021; 9.53 × 8.98 mm; female (ov) 8.83 × 7.78 mm; female 7.65 × 7.22 mm. ***Paratypes***: MUPADI–Crus–02–16, same data as holotype; female (ov) 8.83 × 7.78 mm; female 7.65 × 7.22 mm. SMF57499, West Atlantic, Colombia, Bolívar, Islas del Rosario, Isla San Martín de Pajarales, 10°10.637'N, 75°46.234'W; in coral gravel, 1–2 m, leg. B. Werding, Sep. 2001; female (ov) 7.0 × 6.8 mm; male 7.3 × 7.0 mm; male 7.7 × 7.6 mm; male 5.4 × 5.2 mm; female (ov) 7.7 × 7.4 mm; female (ov) 7.6 × 7.6 mm; male 6.1 × 5.8 mm; female (ov) 7.5 × 7.7 mm; female (ov) 6.4 × 6.5 mm; male 5.4 × 5.3 mm; female 6.1 × 6.2 mm.

##### Other material examined

**(personal collections by the authors).** West Atlantic, Belize, Carrie Bow Cay, 16°48.188'N, 88°5.067'W; under blocks of dead elkhorn coral, 1–2 m, leg. A. Hiller, Jun. 2016; male 5.1 × 4.9 mm; female 5.3 × 5.0 mm.

##### Description.

Carapace slightly longer than broad, evenly rounded along branchial margins, broadest on midbranchial level. Surface with transverse, piliferous plications, one epibranchial spine present. Front sinuously triangular with a longitudinal depression, its borders fringed by a row of spinules, giving a serrated aspect; orbitae moderately deep, supraocular spine strong, postorbital angle produced into a spine-tipped tooth. Eyes moderately large, dorsal extension onto cornea narrow. Basal segment of antennulae with some transverse rugae, anterior margin with teeth. First movable segment of antennae with a serrated, spine-tipped lamellar lobe; second and third segments slightly rugose, flagellum naked.

Chelipeds sub-equal, surface with piliferous striations, merus rugose with serrated, spine-tipped lobe on anterior margin; carpus about two times as long as broad, armed on anterior margin with four, seldom five broad, serrated teeth; posterior margin slightly convex, armed with a row of strong, forwardly directed curved spines. Palm of manus broad with an inconspicuous longitudinal ridge ending at angle between fixed finger and pollex. Lateral surface of dactylus with interrupted transverse, piliferous striations; lateral surface of pollex with rough, conical protuberances extending to fixed finger; outer margin of palm convex, with row of strong, forwardly directed spines, frequently fringed with feathered setae. Gape of fingers with extended ventral pubescence covering proximal portions of pollex and dactylus. Walking legs rugose; anterior margin of merus with fringe of plumose setae, all segments covered with irregularly, wide-set, simple setae; anterior margin of merus with row of spines; large posterodistal spine on merus of walking legs 1 and 2, frequently a smaller one on leg 3; carpus of all walking legs with anterodistal spine; propodus with terminal triplet of movable spines on ventral border, with one or two additional ones; dactylus large, with four movable spinules on inner margin.

Telson seven-plated with a few short, transverse, piliferous ridges.

##### Colouration.

The overall colouration of most specimens consists of a brownish beige background, partly overlaid with iridescent blue tones towards the posterior part of the carapace and on the chelipeds; the transverse ridges of carapace and extremities are marked by blue stripes delineated by narrow dark purple lines. The distal articulations of the walking legs are spotted with blue and purple (Fig. 6a, b). Other specimens show an entirely blue background with similar dark purple delineations of transverse ridges and granules (Fig. 6c).

The new species was first perceived as a different colour morph of *P.caribensis*, as they are not distinguishable through the two main diagnostic traits (Table 1): both bear one pair of epibranchial spines and four movable spines on the dactylus of all walking legs. However, while adult males and females of *P.caribensis* are relatively small, with carapace lengths of up to 9.4 mm in males and 7.5 mm in females, those of *P.coeruleus* sp. nov. reach significantly larger sizes, with carapace lengths of up to 12.4 mm in males, and 12.0 mm in females. The chelipeds in the new species have a more compact aspect, and the dorsal ornamentation on the outer surface of the cheliped’s palm is conspicuously more pronounced than in *P.caribensis*, which only bears scattered low granules (Fig. 8a, b).

##### Ecology.

While *Petrolisthescaribensis* is a typical inhabitant of shallow-water coralline environments, mostly in the dead bases of the finger coral *Poritesporites* (Pallas), *Petrolisthescoeruleus* sp. nov. has a wider habitat spectrum and depth range, as it occurs under boulders in protected sites of the surf zone. The authors found the species on roots of the red mangrove *Rhizophoramangle* L. in a coastal lagoon in the Colombian Gulf of Morrosquillo (9°41.684'N, 75°41.135'W), and also in the furrows of the giant barrel sponge *Xestospongiamuta* (Schmidt, 1870) at 8 m depth in the same locality (Table 2). Sequences of the 16S DNA gene previously published by the authors and deposited in the GenBank database (www.ncbi.nlm.nih.gov/Genbank: sequences published as *P.galathinus* “Blue”, Accession No. DQ444890–DQ444898) match a sequence from the Yucatán Peninsula collected at 20–29m, published as *P.galathinus* by Bracken-Grissom et al. (2013; accession no. KF182548). This record increases the depth range of the new species, which extends from the upper subtidal at 0.5 m to 29 m depth.

**Table 2.** Geographic and ecological range of the species and colour morphs comprising the *Petrolisthesgalathinus* complex and allied species. WA = West Atlantic; EP = East Pacific; FMNH = Florida Museum of Natural History.

Species	Geographic range	Ecology
*Parapetrolisthestortugensis* (Glassell, 1945)	WA: Florida, Bahamas, Gulf of Mexico, Belize, Costa Rica, Panama, Colombia, Venezuela, Antilles (Werding et al. 2003; Poupin and Lemaitre 2014; personal records AH and BW)	Coral rubble, from sponges of the genus *Ircinia* Nardo, 1833; pers. comm. F. Sanford), 0.5–54 m (Haig 1956; Werding et al. 2003; Poupin and Lemaitre 2014; personal records AH and BW)
*Petrolisthesbolivarensis*	WA: Florida, Panama, Colombia, Venezuela (Werding and Kraus 2003; personal records AH and BW)	Dead part of *Poritesporites* (Pallas, 1766), under boulders in protected sites of the surf zone, under dead blocks of *Acroporapalmata* (Lamarck, 1816), 0–1.5 m (Werding and Kraus 2003; personal records AH and BW)
*Petrolisthescaribensis*	WA: Florida, Bahamas, Gulf of Mexico, Belize, Panama, Colombia, Venezuela, Antilles, (Werding 1983; Werding et al. 2003; Poupin and Lemaitre 2014; FMNH portal; personal records AH and BW)	Dead part of *Poritesporites*, under dead blocks of *Acroporapalmata*, 0.5–22 m, on coral heads and rubble in shallow waters at 4 m, on outer reef slope ≤11 m (Werding et al. 2003; Poupin and Lemaitre 2014; personal records AH and BW)
*Petrolisthescoeruleus* sp. nov.	WA: Bahamas, Gulf of Mexico, Belize, Panama, Colombia (Hiller et al. 2006; FMNH portal; personal records AH and BW). Possibly in Florida up to South Carolina (personal records AH and BW; pers. comm. D. Knott)	Dead part of *Poritesporites*, under boulders in protected sites of the surf zone, under dead blocks of *Acroporapalmata*, on roots of *Rhizophoramangle* Linnaeus, 1753, in the furrows of *Xestospongiamuta* (Schmidt, 1870), 8 m (Quiceno–Cuartas 2012); 0.5–29 m (Bracken–Grissom et al. 2013; personal records AH and BW)
*Petrolisthescolumbiensis*	WA: Colombia, Cuba (Werding et al. 2003)	Dead part of *Poritesporites*, 1–6 m (Werding et al. 2003; Hiller et al. 2006; personal records AH and BW)
*Petrolisthesgalathinus* “Stripes–Spots”	WA: Panama, Colombia, Guyana, Brazil (Hiller et al. 2006; personal records AH and BW)	Dead part of *Poritesporites*, in reef of *Agaricia* Lamarck, 1801, under boulders in protected sites of the surf zone, 0.5–3 m (Hiller et al. 2006; personal records AH and BW)
*Petrolisthesgalathinus* “White Teeth”	WA: Florida, Gulf of Mexico, Belize, Panama, Colombia, Venezuela, Antilles, possibly along the east coast of Florida (Hiller et al. 2006; Rodríguez et al. 2006; Poupin and Lemaitre 2014; personal records AH and BW)	Dead part of *Poritesporites*, under dead, large blocks of *Acroporapalmata*, under boulders in protected sites of the surf zone, on coral heads, 0.5–6 m (Hiller et al. 2006; Poupin and Lemaitre 2014; personal records AH and BW)
*Petrolisthesoccidentalis*	EP: Mexico (Cuastecomate Bay, Jalisco), El Salvador, Costa Rica, Panama, Colombia, Ecuador (Haig 1960; Moran 1984; Hiller et al. 2004, 2006; Ferreira and Anker 2021; personal records AH and BW)	Under boulders at 0–2 m; dredged from sand and sand-shell bottoms at 7.2 and 18 m; among rocks with oysters (Haig 1960; Moran 1984; Hiller et al. 2004; personal records AH and BW)
*Petrolisthesrosariensis*	WA: Bahamas, Gulf of Mexico, Belize, Panama, Colombia, Venezuela, Antilles, Brazil (Werding et al. 2003; Poupin and Lemaitre 2014; FMNH portal; personal records AH and BW)	Dead part of *Poritesporites*, under dead, large blocks of *Acroporapalmata*, under boulders in protected sites of the surf zone, on coral heads, in *Agaricia* coral reef framework, under boulders (Hiller et al. 2006; FMNH portal); 0.5–35 m (Poupin and Lemaitre 2014; personal records AH and BW)
*Petrolisthessanmartini*	WA: Bahamas, French Antilles, Colombia (Werding and Hiller 2002; Werding et al. 2003; FMNH portal; personal records AH and BW)	Dead part of *Poritesporites*, in coral rubble; subtidal to 18 m (Werding and Hiller 2002; Werding et al. 2003; FMNH portal; personal records AH and BW)

##### Distribution.

*Petrolisthescoeruleus* sp. nov. is, so far, known from the Colombian and Panamanian Caribbean, Belize, and the east coast of Mexico. R. Lasley (FMNH; pers. comm. Nov. 2021) confirmed the species to be present in the Bahamas as well. Also, through D. Knott (PoseIDon Taxonomic Services, LLC, Charleston, South Carolina, U.S.A.; pers. Comm. Oct. 2021) we became aware of material of *P.galathinus* collected in South Carolina by SERTC (Southeast Regional Taxonomic Center, South Carolina). Part of the specimens exhibit four spines on the dactylus of the walking legs, thus opening the possibility that the distribution of the new species may reach northern waters along the U.S. western coast. Collections on the coast of South Carolina await further examination to confirm if the new species indeed reaches this northern locality.

##### Etymology.

The name *coeruleus* alludes to the blueish tone of carapace and extremities, which comprises a reliable diagnostic character to distinguish this species from *P.caribensis*.

#### ﻿ Petrolisthes occidentalis

Taxon classificationAnimaliaDecapodaPorcellanidae

Stimpson, 1859

BE8865B0-D19A-512D-BF56-345D3729C7A7

Fig. 5a

Petrolisthes occidentalis Stimpson, 1858: 227 (nomen nudum; listed); Stimpson 1859: 73 (description); Streets 1871: 240; Lockington 1878: 395; Faxon 1893: 175; Faxon 1895: 69; Ferreira and Anker 2021: 107.
Petrolisthes galathinus Ortmann, 1897: 284; Schmitt 1935: 186; Haig 1960: 36; Haig 1962: 176 (part); Gore and Abele 1976: 21; Gore 1982: 13; Moran 1984: 78; Hiller et al. 2004: 5; Hiller et al. 2006: 548.

##### Type material examined.

***Syntypes***: MCZ:IZ:CRU–1401, East Pacific, Panama, 1 male, 3 females (ov).

##### Other material examined.

MUPADI–Crus–02–17, East Pacific, Panamá, Panama City, Punta Culebra, under large boulders, low intertidal, leg. A. Hiller, 21 Feb. 2015; male, 12.8 × 13.5 mm; male, 12.9 × 13.4 mm.

##### Diagnosis.

*Petrolisthesoccidentalis* morphologically resembles the other members of the *P.galathinus* complex. An extensive description was given by Haig (1960). The species shares with most members of the group the presence of one epibranchial spine and three movable spines on the ventral side of the dactylus of the walking legs. *P.caribensis*, *P.columbiensis*, and *P.coeruleus* sp. nov. bear four such spines, and *P.sanmartini* bears five (see Table 1). The borders of the carpus of the chelipeds tend to be subparallel in *P.occidentalis* giving the carpus a straight and slender look. In the Atlantic forms, the anterior margin is more convex. *Petrolisthesoccidentalis* reaches larger sizes than all Atlantic forms, with carapace lengths of more than 16 mm.

##### Colouration.

The transverse ridges and tubercles, which are typical of the members of the *Petrolisthesgalathinus* species complex, are bordered with purplish red bands, the intervening grooves are yellowish, the yellow colour prevailing on the carpus teeth. The merus of the walking legs is irregularly spotted with purplish dots, the carpus and propodus show three broad, purplish bands alternating with paler yellowish ones (Fig. 5a).

##### Remarks.

*Petrolisthesoccidentalis* was listed by Stimpson (1858), and one year later it was described by the same author (Stimpson 1859) from the Pacific coast of Panama. In the description he stated that the species is “scarcely to be distinguished from *P.sexspinosus* Stimpson, 1858”, an older synonym of the western Atlantic *P.galathinus*. In the decades after Stimpson´s studies, various authors referred to the Pacific populations as *P.occidentalis* (see Streets 1871; Faxon 1893, 1895). Ortmann (1897) emphasised the lack of differences between specimens from western India and Pacific Panama, stating that since *P.galathinus* occurred likewise on the east and west of tropical America, *P.occidentalis* should be treated as a synonym of *P.galathinus* (arguments reviewed by Haig 1960).

Hiller et al. (2006) postulated that the Eastern Pacific *P.galathinus* deserves specific status, given the relatively large genetic distances between the Pacific and the most closely related Atlantic clades, which comprised the “Spots” and “Striped” morphs. The East Pacific species differs in colour and size from all Atlantic forms. In a local catalogue of the Porcellanidae of Panama, Ferreira and Anker (2021) published a short note suggesting the need to resurrect the species, based on the recommendations by Hiller et al. (2006).

##### Geographic range.

Haig (1960) highlighted the discontinuous distribution of the species in the East Pacific, with a concentration near Panama City, and only few findings from Isla San Lucas, Costa Rica, and off La Libertad, Ecuador. Based on new records of the species in the Panamanian and Colombian East Pacific, Hiller et al. (2004) confirmed that *P.occidentalis* seems to have a continuous distribution from Jalisco, south side of Cuastecomate Bay, Mexico, throughout Central America and Colombia, reaching Salinas and La Libertad in Ecuador.

## ﻿Discussion

For more than two centuries *Petrolisthesgalathinus* has been viewed as a widely dispersed species in the West Atlantic and East Pacific, with a broad ecological range, inhabiting a variety of substrates like rocks, corals, and sponges, and from the upper subtidal down to 50 m depth. This species turned out to be a complex of morphologically similar species; so far, the *P.galathinus* complex encompasses eight species, including *P.coeruleus* sp. nov. and the resurrected *P.occidentalis*. Each species is supported by distinctive mitochondrial DNA sequences, colouration, adult size, larval morphology, and shape of the sternal plate. All species, except *P.rosariensis*, share a most recent common ancestor (MRCA), which started diverging into different Atlantic and Pacific lineages before the Central American Isthmus finished rising and interrupted gene flow between populations from each ocean (Hiller et al. 2006).

*Petrolisthesgalathinus* still needs more revision, as it is unclear which of the molecular clades, designated in the phylogeny by Hiller et al. (2006) as “White Teeth”, “Stripes”, and “Spots”, corresponds to Bosc´s (1802) description. The three variants have different geographic distributions, overlapping in the southern Caribbean. However, while the “Stripes” and “Spots” morphs appear to have a southern distribution reaching Brazil (Hiller et al. 2006), the “White Teeth” morph extends to the Gulf of Mexico, up to the Florida Keys (pers. obs.). Since the “White Teeth” morph extends its range from the southern Caribbean to Florida, it is probable that this is the morph reported from Cape Hatteras, North Carolina by Haig (1960).

The *P.galathinus* complex poses an interesting case to study speciation within allopatric and sympatric scenarios, as it comprises closely related species on either side of the Isthmus of Panama. Such a unique assemblage allows assessing the relationship between genetic divergence and reproductive isolation, given the background of a relatively recent and well dated geological barrier that resulted in sister lineages on each side of the Americas (Hiller and Lessios 2017, 2019).

The evolutionary, ecological, and geographic processes that gave rise to the formation of different West Atlantic species with similar geographic ranges and ecologies remains to be explained in the light of a multigene phylogeographic approach of the species complex. The overlapping geographic and ecological ranges in the southern Caribbean (Table 2) are suggestive of ecological speciation driven by different microhabitats offered by coral reefs, where all species occur.

## Supplementary Material

XML Treatment for Petrolisthes coeruleus

XML Treatment for Petrolisthes occidentalis

## ﻿Additional information

### Author ORCIDs

Alexandra Hiller https://orcid.org/0000-0001-9812-2126

Bernd Werding https://orcid.org/0000-0002-1311-6139

### Data availability

All of the data that support the findings of this study are available in the main text.
